# Annual variations in the number of malaria cases related to two different patterns of *Anopheles darlingi *transmission potential in the Maroni area of French Guiana

**DOI:** 10.1186/1475-2875-9-80

**Published:** 2010-03-22

**Authors:** Florence Fouque, Pascal Gaborit, Romuald Carinci, Jean Issaly, Romain Girod

**Affiliations:** 1 Institut Pasteur, Cellule d'Intervention Biologique d'Urgence (CIBU), 25-28, rue du Dr Roux, 75724 Paris cedex 15, France; 2 Unité d'Entomologie Médicale, Institut Pasteur de Guyane, 23, Avenue Pasteur, BP 6010, 97300 Cayenne, French Guiana

## Abstract

**Background:**

With an Annual Parasite Incidence (API) of 132.1, in the high and moderate risks zones, the Maroni area of  French Guiana has the second highest malaria incidence of South-America after Guyana (API = 183.54) and far above Brazil (API = 28.25). Malaria transmission is occurring despite strong medical assistance and active vector control, based on general WHO recommendations. This situation is generated by two main factors that are the social and cultural characteristics of this border area, where several ethnic groups are living, and the lack of understanding of transmission dynamics of the main mosquito vector, *Anopheles darlingi.* In this context, entomological data collected in two villages belonging to two different ethnic groups of the French border of the Maroni River, were retrospectively analysed to find out how the mosquito bionomics are related to the malaria transmission patterns.

**Methods:**

Data were provided by human landing catches of mosquitoes carried out each month for two years in two villages belonging to two ethnic groups, the Amerindians Wayanas and the Aloukous of African origin. The mosquitoes were sorted by species, sex, date, hour and place of collection and processed for *Plasmodium sp*. parasite detection. The data were compiled to provide the following variables: human biting rates (HBR), parity rates (PR), numbers of infective bites (IB), entomological inoculation rates (EIR) and numbers of infected mosquitoes surviving enough to transmit (IMT). Spatial and temporal differences of variables between locations and during the night were tested by the Kruskall-Wallis analysis of variance to find out significant variations.

**Results:**

The populations of the main mosquito vector *An. darlingi *showed significant variations in the spatial and temporal HBR/person/night and HBR/person/hour, IB/person/month and IB/person/hour, and IMT/village/night and IMT/village/hour. In the village of Loca (Aloukous), the IMT peaked from June to August with a very low transmission during the other months. The risks were higher during the first part of the night and an EIR of 10 infective bites per person and per year was estimated. In the village of Twenke (Wayanas), high level of transmission was reported all year with small peaks in March and October. The risk was higher during the second part of the night and an EIR of 5 infective bites per person and per year was estimated.

**Conclusion:**

For the first time in the past 40 years, the mosquito bionomics was related to the malaria transmission patterns in French Guiana. The peak of malaria cases reported from August to October in the Maroni region is concomitant with the significant peak of *An. darlingi *IMT, reported from the village of Loca where transmission is higher. However, the persistent number of cases reported all year long may also be related to the transmission in the Amerindian villages. The *An. darlingi *bionomics for these two close populations were found significantly different and may explain why a uniform vector control method is inadequate. Following these findings, malaria prevention measures adapted to the local conditions are needed. Finally, the question of the presence of *An. darlingi *sub-species is raised.

## Background

With about 4,000 cases of malaria annually [1] and an annual parasite incidence (API) of 132.1 in the high and moderate risks zones, the Maroni area of French Guiana has the second highest malaria incidence of South-America, after Guyana (API = 183.59) and far higher than Brazil (API = 25.23) [2]. Malaria transmission is reported mostly from the Maroni and Oyapock regions with almost no transmission in the main city of Cayenne and low number of cases in the coastal areas [3,4]. A first control of the disease was successfully maintained between the 1950s and the 1970s, due to DDT sprayings [5]. Plasmodium indices dropped from 26.1% in 1949 to 0.9% in 1952 [6-8]. Then, malaria incidence increased from 117 cases and an incidence of 2.3 per 1,000 in 1970 [7] to 3,349 cases and an incidence of 38.5 per 1,000 in 1987 [9]. A peak of about 6,000 malaria cases was reached in 1989 and again in 1995 [10]. Until the year 2005, 90% of the malaria cases were due to *Plasmodium falciparum *[9-11], but then a change was reported with 50% of the cases due to *Plasmodium vivax *[11,12]. The possible reasons for such an increase in the malaria incidence are numerous, such as mosquito resistance to insecticides, parasite resistance to anti-malarial drugs, population movements due to political reasons or gold mining, but also changes in the biting behavior of the main mosquito vector *Anopheles (Nyssorhynchus) darlingi *[3,5,10].

Since the first studies on the vectors of malaria in French Guiana, the species *An. darlingi *was reported as the main and sometimes the exclusive vector [6,13,14]. *Anopheles darlingi *is widely distributed in different ecological systems of French Guiana [14,15]. Other species, such as *Anopheles (Nyssorhynchus) aquasalis*, *Anopheles (Nyssorhynchus) braziliensis*, *Anopheles (Nyssorhynchus) nuñez-tovari, Anopheles (Kerteszia) neivai *and *Anopheles (Nyssorhynchus) oswaldoi *are also present in French Guiana [15]. The bionomics of *An. darlingi *in the coastal areas of French Guiana were studied during the late 1970s and showed an exophilic biting preference with tri-modal biting peaks around 6:00 PM, 1:00 AM to 2:00 AM and around sunrise at 7:00 AM [13]. However, very little is known about the indoor resting behaviour of *An darlingi *and the few studies that have been done report all night indoor resting in Brazil [16] to only two to three hours indoor resting in Surinam [17]. Recent entomological investigations in two different areas of the Maroni River reported a great behavioural heterogeneity, but no relationship could be found between *An. darlingi *parameters and malaria transmission [18,19].

The "Service Départemental de Démoustication" (SDD) is in charge of malaria prevention and vector control in French Guiana. Malaria cases detection is based on examination of blood smears from febrile patients and vector control consists of chemical spraying of houses and dependences [3,4,11,14]. Some impregnated bed-nets campaigns are attempted regularly, but do not reached the required level in the populations at risks. Because the data available on the bionomics of the malaria vector *An. darlingi *inside the Amazonian forest of French Guiana, such as the biting activity and infection rate, could not be related to malaria transmission [18,19], it appears very difficult to decide which vector control measures are best adapted to the situation. Furthermore, the populations living in the Maroni region have strong ethnic, cultural and social differences, and a unique vector control strategy may not be applicable to every local situation. In this context, a retrospective analysis of entomological data collected during long-term studies of the malaria transmission risks carried out along the Maroni River is presented. The data were collected in two locations each with populations belonging to two different ethnic groups. Biting rates, parity and transmissions risks for *P. falciparum*, *P. vivax *and *P. malariae *were determined monthly over a two-year period in an "African-type" village and an Amerindian village. The results are then discussed according to recently published malaria transmission data and vector control options.

## Methods

### Study sites

**Figure 1:**
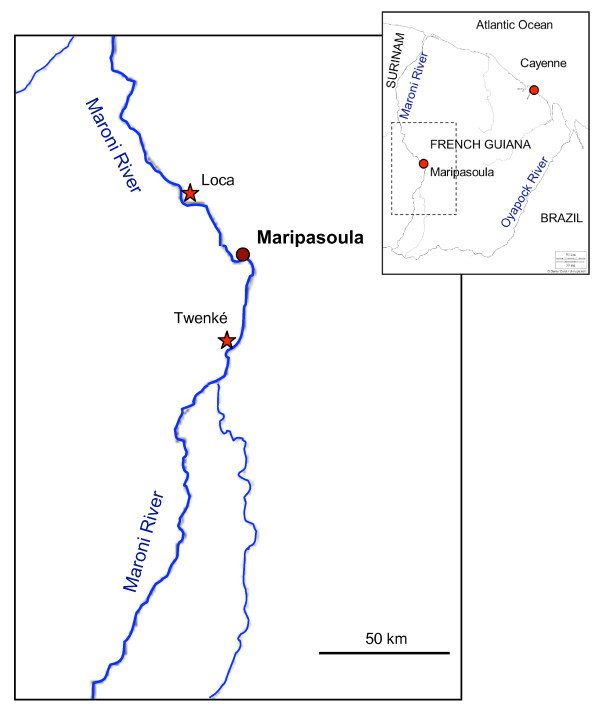
**Study sites**. Simplified map of the upper Maroni River (in dark blue) with the Aloukou village of Loca (north of the main town of Maripasoula), and the Amerindian village of Twenke (south of Maripasoula).

The mosquito collections were performed in Loca and Twenke villages situated along the Maroni River (Figure 1). The village of Loca (latitude 3°49'27"N, longitude 54°11'33"W) is located at about two hours by boat and 30 km north from the main city of Maripasoula and has a population belonging to the ethnic group of Bonis/Aloukous, an African-type population, escaped from Dutch slavery two centuries ago. The village is composed of about 30 houses and is inhabited by about 100 persons, mostly women and children because the men are often working outside. The housing in Loca is heterogeneous with modern houses and old-style houses with disjointed woodcut. The village of Loca is known as a *P. falciparum *malaria focus. The village of Twenke (latitude 3°22'15"N, longitude 54°03'35"W) is situated at about 30 km south of Maripasoula (Figure 1), in an area inhabited by Amerindians belonging to the Wayanas ethnic group. The village has about 20 houses and is inhabited by about 150 persons from all ages who live in family groups. The housing of this village is traditional Amerindian with wooden stilts houses one storey high, no walls in the basement, and sometimes just two walls on the first floor. The village of Twenke is a malaria focus where both *P. falciparum *and *P. vivax *have been reported.

### Mosquito captures

Human landing catches [20] were carried out in each location by two persons from 6:30 PM to 0:30 AM, and 2 persons from 0:30 AM to 6:30 AM. The teams were regularly rotated to avoid collection bias. The starting and ending collection hours were chosen according to the dusk and sunshine hours. The collections were made outside in the verandas of schools and/or community houses, having no people at night and situated in the middle of the villages. The collections were performed monthly during two consecutive nights in each village, beginning in March 1998 and ending in December 1999.

### Treatment of mosquitoes

Females were identified and counted by species and sex the following morning with a field stereoscope. For each location, species, and hour, 10 females (or less) were dissected and the ovaries observed to determine the parity rates [21]. Mosquitoes were held desiccated individually into micro tubes with a record of species, sex and time of capture for later processing to determine their infection status. The head and thorax of each mosquito was then placed in a single tube, triturated in PBS and centrifuged at 3,000 rpm. The supernatant was then placed in an ELISA plate to detect the presence of *Plasmodium *antigen in the salivary glands. The *Plasmodium *detection by ELISA was based on the circumsporozoite protein detection [22] for the three *Plasmodium *species reported from the areas under survey. The following four strains of *Plasmodium *were tested: one strain of *P. falciparum*, two strains of *P. vivax, (P. vivax*-210 and *P. vivax*-247) and one strain of *P. malariae*.

### Estimation of the numbers of infected mosquitoes surviving enough to transmit

The survival of the females collected under field conditions was estimated from the monthly mean proportion of parous females according to the formula using the gonotrophic cycle mean duration [23,24]. Very few data are available on the duration of the gonotrophic cycle of *An. darlingi*. An average value of 2.3 days was obtained under field conditions in Brazil [25], but the gonotrophic cycle duration can vary from 2.4 to 4.4 days for the same species and from 2 to 5 days for *Nyssorhynchus *species [26]. Unpublished laboratory observations showed that the gonotrophic cycle of *An. darlingi *in French Guiana varied between three and four days. Thus a mean value of 3.5 days was considered a good approximation. The survival estimates were then used to calculate the number of infected mosquitoes able to transmit (IMT), that is having a life duration longer than the duration of the extrinsic amplification. This IMT number was derived from the entomological parameters of the basic case reproductive rate defined by MacDonald [27] and used for vectorial capacity as developed by Garrett-Jones *et al *[28]. The IMT number is calculated according to this simple equation:

and was chosen because it can be estimated with only four variables, including the HBR [28], the survival (p) [27], the proportion of infected mosquitoes (b) and the duration of extrinsic incubation (n) [27,29,30]. The first three variables were available from the field data and the estimation of b was a mean annual value for each location. The duration of the extrinsic incubation period (EIP) of the *Plasmodium species *in the mosquito *An. darlingi *has not been investigated, thus this variable was estimated through a logarithmic function with data reported for the sporogonic cycle of *P. falciparum *according to different temperatures [31,32]. The final value of the IMT was estimated per village on the base of 100 people in each village.

### Statistical analysis

Continuous data were collected and to avoid bias in the distribution of the data into artificial classes, the normality of the data was not tested. The significance of the inter-groups differences among monthly and hourly variables was tested for each location with a non-parametric Analysis of Variance (ANOVA) according to the Kruskal-Wallis test with tied ranks [33]. The single factor analysis of variance was chosen because of unequal cell sizes [34]. Ranking of the values was made according to the Mann-Whitney test with tied ranks [35]. The statistical analyses were performed per month and per hour on the human biting rates (HBR), the parity rates (PR), the numbers of infective bites (IB) and the IMT. Raw data were used for monthly comparisons and monthly means were used for hourly comparisons.

### Climatic data

The mean monthly rainfalls, maximum and minimum temperatures reported for the nearest town of Maripasoula (Figure 1) were provided by the National Oceanic and Atmospheric Administration (NOAA) through their website. The mean monthly flows of the Maroni River at Maripasoula, observed between 1953 and 2003, were extracted from the unpublished report "Régime hydrologique des fleuves guyanais: Etude fréquentielle des débits" provided by the Direction de l'Environnement from French Guiana.

### Epidemiological data

The data were provided by the Institute of Health Surveillance (InVS) [11].

### Ethics

The methodology for the mosquito captures was approved by the ethical committee of the Direction Départementale des Affaires Sanitaires et Sociales (DDASS) of French Guiana, representing the French Ministry of Health. The persons collecting mosquitoes were legally appointed by the Institut Pasteur of French Guiana and had salaries and access to all health care protection provided to the Institut Pasteur dependents.

## Results

### Annual variations of the Human Biting Rates (HBR)

**Figure 2:**
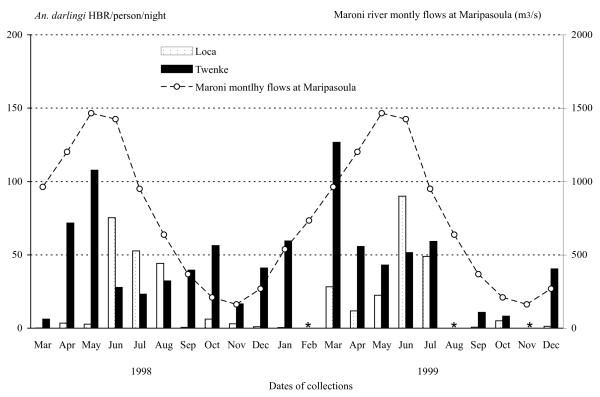
**Field HBR/person/nigh**. *Anopheles darlingi *HBR/person/night (Human Biting Rates) estimated from the field collection in Loca and Twenke, from March 1998 to December 1999, and fluctuations of the means monthly flows (in m^3^/second) of the Maroni River at Maripasoula. No captures were done for the months of February, August and November 1999 (*).

**Figure 3:**
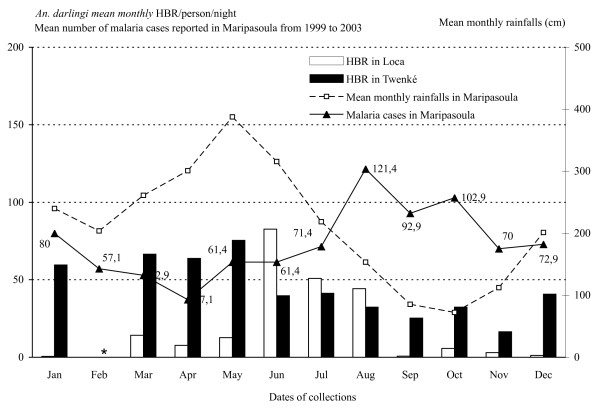
**Monthly means of HBR/person/nigh**. Monthly means of *An. darlingi *HBR/person/night (Human Biting Rates) in Loca and Twenke, and fluctuations of the monthly means rainfalls in Maripasoula, and the monthly mean numbers of malaria cases reported from Maripasoula. No captures were done for the months of February (*).

From March 1998 to December 1999, 42 field trips were carried out in the villages of Loca and Twenke. The collections included 83 nights and 166 nights-men captures, and yielded a total of 5,187 Anopheles. The detailed data are reported in the Additional file 1. The captures gathered 5,182 (99.9%) *An. darlingi*, three *An. nuñez-tovari *(0.05%), one *Anopheles (Anopheles) intermedius *(0.02%) and one non-identified *Anopheles *species. In the two years of the study, almost twice as many *An. darlingi *were captured in Twenke (3,590) than in Loca (1,597). The HBR of *An. darlingi *was highly variable during the year with a marked seasonality (Figure 2). In Loca, the collections yielded a mean number of 20.17 bites/person/night and an annual 8,126 bites/person/year, with a minimum of 0.13 females/person/night in March 1998 and a maximum of 90 females/person/night in June 1999. In Twenke, the collections yielded a mean number 46.18 bites/person/night and an annual 16,355 bites/person/year, with a minimum of 6.13 females/person/night in March 1998 and a maximum of 126.75 females/person/night in March 1999. The patterns were different between the two villages and also between the two years (Figure 2). In Loca, *An. darlingi *HBR peaked significantly in June, July and August (Table 1). In Twenke, mosquitoes where collected in high number all year with important year to year variation, the highest values were reported in April and May 1998 and in March 1999 with no significant peaks (Table 1). No data were available on the rainfalls in the locations of Loca and Twenke, but the mean monthly rainfalls reported from Maripasoula (Figure 1) shows that the mean monthly mosquito numbers were found around the rainy season, in June in Loca and from March to May in Twenke, and the lowest mean monthly numbers were found during the dry season from September to November (Figure 3). Furthermore, the peaks of HBR found from March to June could be linked to the Maroni River level (Figure 2). The *An. darlingi *HBRs were thus varying with the rainfalls and the Maroni River water level. The mean monthly numbers of malaria cases reported in Maripasoula for the years 1999 to 2003 [11] show that malaria transmission peaks from August to October, i.e. at the beginning of the dry season when the HBRs are at their lowest values (the lowest HBRs are found from September to January in Loca and from September to November in Twenke), and has a minimum in April at the beginning of the rainy season (Figure 3).

**Table 1:** Results of the Kruskal-Wallis Analysis of Variance with tied ranks.

Variables	Location	N	H	Sum T	C	Ho	DF	Chi-2, 0.05	P	Result
Monthly means HBR/person/night	Loca	38	31.94	450	0.9918	**32.20**	10	**18.307**	0.005<P < 0.01	**Reject Ho**
	Twenk	41	13.79	18	0.9997	13.79	10	18.307	0.005<P < 0.01	Do not reject Ho

Monthly means PR	Loca	28	11.01	406	0.9978	11.03	9	16.919	0.005<P < 0.01	Do not reject Ho
	Twenk	40	13.34	30	0.9995	13.34	10	18.307	0.005<P < 0.01	Do not reject Ho

Means HBR/person/hour	Loca	24	13.41	18	0.9987	13.43	11	19.675	0.005<P < 0.01	Do not reject Ho
	Twenk	24	21.94	6	0.9996	**21.95**	11	**19.675**	0.005<P < 0.01	**Reject Ho**

Means PR/hour	Loca	24	6.46	24	0.9983	6.47	11	19.675	0.005<P < 0.01	Do not reject Ho
	Twenk	24	10.24	101	0.9927	10.31	11	19.675	0.005<P < 0.01	Do not reject Ho

Monthly means IB/person	Loca	38	31.94	114	0.9979	**32.00**	10	**18.307**	0.005<P < 0.01	**Reject Ho**
	Twenk	41	13.46	18	0.9997	13.79	10	18.307	0.005<P < 0.01	Do not reject Ho

Monthly means IMT/night/village	Loca	38	31.74	18	0.9997	**31.75**	10	**18.307**	0.005<P < 0.01	**Reject Ho**
	Twenk	41	11.14	0	1.0000	11.14	10	18.307	0.005<P < 0.01	Do not reject Ho

Means IB/person/hour	Loca	24	13.41	18	0.9987	13.43	11	19.675	0.005<P < 0.01	Do not reject Ho
	Twenk	24	21.95	6	0.9996	**21.95**	11	**19.675**	0.005<P < 0.01	**Reject Ho**

Means IMT/village/hour	Loca	24	18.30	0	1.0000	**18.30**	11	19.675	0.005<P < 0.01	Do not reject Ho
								**17.275**	0.01<P < 0.025	**Reject Ho**
	Twenk	24	21.34	0	1.0000	**21.34**	11	**19.675**	0.005<P < 0.01	**Reject Ho**

Results of the Kruskal-Wallis Analysis of Variance with tied ranks for the following variables: the Monthly means HBR/person/night, the monthly means PR, the means HBR/person/hour, the means PR/hour, the monthly means IB/person, the monthly means IMT/night/village, the means IB/person/hour and the means IMT/village/hour.

### Annual variations of parity rates (PR)

**Figure 4:**
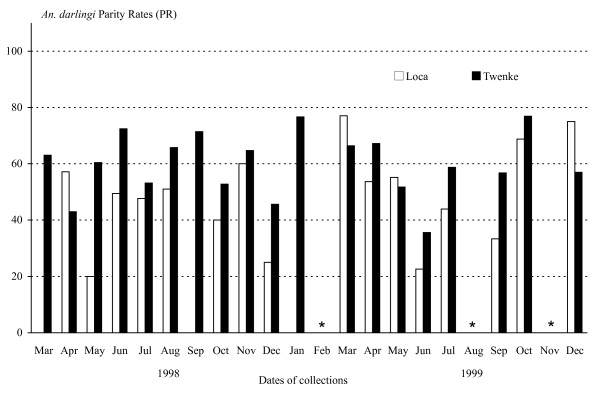
**Field Parity Rates**. *Anopheles darlingi *PR (Parity Rates) estimated from the field collections in Loca and Twenke from March 1998 to December 1999. No captures were done for the months of February, August and November 1999 (*).

**Figure 5:**
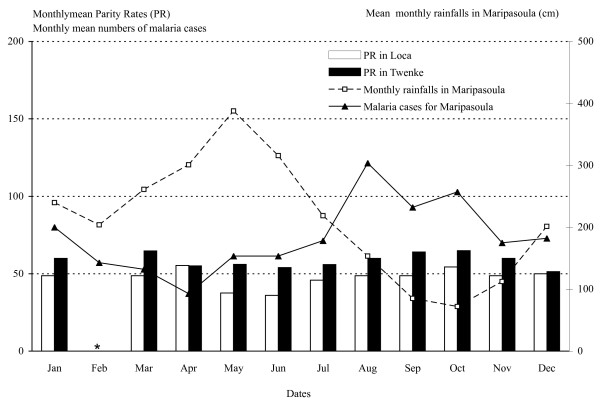
**Monthly means Parity Rates**. Monthly means *An. darlingi *PR (Parity Rates) in Loca and Twenke, and fluctuations of the mean monthly rainfalls in Maripasoula and the mean monthly number of malaria cases reported from Maripasoula. No captures were done for the months of February (*).

A total of 2,713 females of *An. darlingi *were dissected and the parity rates fluctuated between locations and years (Figure 4). In Loca, the monthly mean parity rate was 48.73%, varying from 20% to 77.03%. In Twenke, the monthly mean parity rate was 59.96%, varying from 35.56% to 76.92%. The percentage of parous females is a function of the mosquito survival under natural conditions. Although, no significant variations were estimated (Table 1), the female survival seems a little higher at the beginning of the year for both locations, slightly drops during the rainy season to reach a minimum in May-June, and increases again in August during the dry season (Figure 5).

### Night biting activity

**Figure 6:**
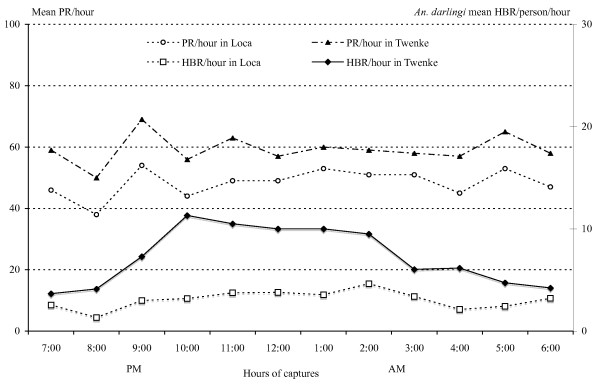
**Means HBR/person/hour and PR/person/hour during one night**. *Anopheles darlingi *mean HBR/person/hour (Human Biting Rates) and PR/person/hour (Parity Rates) during one night in Loca and Twenke.

The mean HBRs/person/hour show different patterns in the two villages (Figure 6). In Loca, a mean number of 1.54 bites/person/hour was reported, varying from 0.67 bites/person/hour to 2.32 bites/person/hour. The mosquito aggressiveness increases slowly during the first part of the night until 2:00 AM, however the peaks were not significant (Table 1). In Twenke, a mean number of 3.65 bites/person/hour was reported, varying from 1.83 bites/person/hour to 5.66 bites/person/hour. The mosquito aggressiveness increases rapidly and significantly during the first part of the night and reaches a peak at 10:00 PM (Table 1, Figure 6). The parity rates of the females were also fluctuating during the night but the patterns were completely similar in both locations, with a lowest percentage of parous females in Loca all night (Figure 6). The peaks of parity observed at 9:00 PM and 5:00 AM were not significant (Table 1).

### Mosquito infection rates and Entomological Inoculation Rates (EIR)

**Figure 7:**
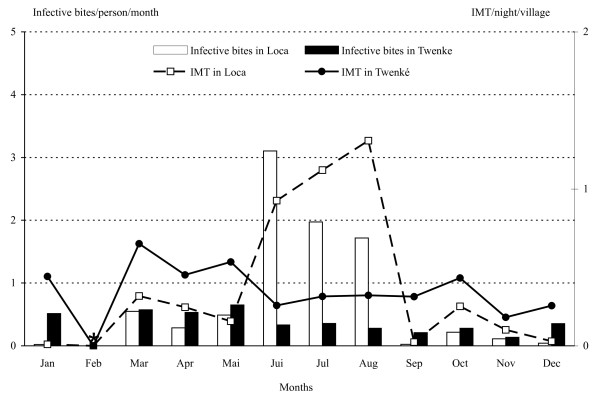
**Monthly means of IB/person and IMT/night/village**. *Anopheles darlingi *monthly means of IB/person (Infective Bites) and of IMT/night/village (Infected Mosquitoes surviving enough to Transmit) in Loca and Twenke. No data were available for the months of January and February in Loca, and February in Twenke (*).

**Figure 8:**
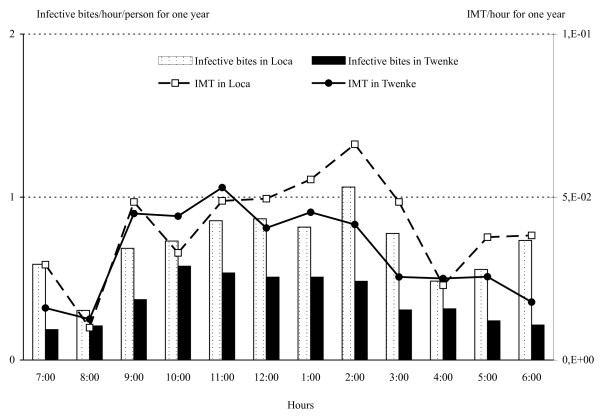
**IB/person/hour and IMT/hour/village during one year**. *Anopheles darlingi *numbers of IB/person/hour (Infective Bites) and of IMT/village/hour (Infected Mosquitoes surviving enough to Transmit) during one year in Loca and Twenke.

The 5,187 *An. darlingi *collected were tested for their infection status and three *P. falciparum *strains were detected, two from Loca and one from Twenke. The *P. falciparum *reported from Loca were found in females collected between 1:30 AM and 2:30 AM on the 16^th ^July 1998, and between 8:30 PM and 9:30 PM on the 24^th ^of April 1999. The strain from Twenke was reported from a female collected between 5:30 AM and 6:30 AM on the 10^th ^of July 1999. The overall infection rate was 0.58/1000 with an infection rate of 1.25/1,000 in Loca and 0.28/1,000 in Twenke. The entomological inoculation rate (EIR) was 10 infective bites/person/year and 5 infective bites/person/year, in Loca and Twenke, respectively. The mean numbers of infective bites/person during the year varies according to the number of bites (Figure 7) with a significant peak from June to August in Loca (Table 1). High numbers of infective bites were found all year in Twenke (Figure 7) with a non-significant peak from March to May (Table 1). The hourly patterns observed during the night are also based on the biting numbers (Figure 8) with a non-significant peak at 2:00 AM in Loca and a significant peak between10:00 PM and 2:00 AM in Twenke (Table 1). For both locations, it is worthwhile to note that the number of infective bites was multiplied by three during the peak.

### Numbers of infected Mosquitoes surviving enough to Transmit (IMT) and transmission risks

The *An. darlingi *IMT fluctuated greatly and significantly monthly and hourly in both villages (Figures 7 and 8, Table 1). In Loca, the IMT peaks significantly in August and during the second part of the night between 1:00 AM and 3:00 AM (Figures 7 &8). The transmission risk increases slowly after 9:00 PM until 2:00 AM and decreases until the morning. In Twenke, the IMT peaks in March and October (Figure 7) and significantly the first part of the night, between 9:00 PM and 2:00 AM (Figure 8). In Twenke, the transmission risk increases rapidly during the first part of the night and remains high for a few hours, than decreases slowly the second part of the night. The higher transmission risk patterns for both Loca and Twenke during the second part of the year is in agreement with the mean number of malaria cases reported by the Health Center of Maripasoula (Figure 5).

## Discussion

Malaria transmission in French Guiana occurs mostly along the Maroni River (Figure 1) from where 77.2% of the cases were reported between 1999 and 2003. The region of Maripasoula represents the second most important focus with 28.5% of cases [11]. The transmission peaks from August to October and 44% of the cases are reported from July to October. The peak of malaria cases occurs during the dry season when *An. darlingi *HBR are low (Figure 2) as already observed in the upper Amerindian villages [18] and the lower Maroni basin [19] but also in the western Brazilian Amazon [36]. At the opposite, the peak of malaria cases reported during the same months in the Venezuelan Amazonian region [37] is concomitant with the rainy season and the peaks of mosquito biting numbers [38]. These observations indicate that Anopheline HBR is fluctuating with the rains or the river levels [39,40] and may not be the most important factor in the malaria transmission risk. In all locations, it is interesting to note that the peaks of malaria cases are found during the hottest months.

The mean entomological inoculation rate (EIR) of about 10 infected bites per person and per year in Loca is in the range of the EIR reported in the same area [19], but the value found in the Amerindian village is lower than reported before [18], that may indicate strong annual variations. In the other Amazonian countries, EIR has a comparable mean value of 10 infective bites/person/year in Rondônia (Brazil) [41] and in Venezuela [37]. But, in those countries, the EIR is also varying from 1.65 positive bites/person/per in Roraima (Brazil) [42] to 129 infective bites/person/year in Upper Orinoco (Venezuela) [38].

*Anopheles darlingi *is one of the most important malaria vector in South-America [43] and the most important vector in the Amazons [44]. Numerous studies were conducted on the population biology and genetics of *An. darlingi *showing the great heterogeneity between populations from different countries for both important behavioral determinants [45] and genetic markers [46,47]. The biting behaviour is varying from uni-modal to tri-modal and biting peaks were found for almost each hour between 6:00 PM and 6:00 AM [43]. When malaria control relies on protecting the people from mosquito bites, it appears impossible to give general recommendations against the species *An. darlingi*. As an example, the impregnated bed-nets will give little protection when the biting peak is between 7:00 PM and 9:00 PM as in Bolivia [48] or between 8:00 and 9:00 PM as in Peru [49], and at the opposite will be very useful when the biting peak is at 11:00 PM as in Surinam [17], at 1:00 AM as in Venezuela [38], or between 1:00 AM and 2:00 AM as in coastal French Guiana [13]. Moreover, all mosquito populations under comparisons were originated from locations separated by long distances, and the genetic differences in such populations can be attributed to a geographical isolation [50]. In this study, the *An. darlingi *populations are almost sympatric with no natural barriers between the populations (Figure 1), and nevertheless show different bionomics patterns. The monthly and hourly biting patterns and even the malaria transmission risk periods are significantly different (Table 1). No genetic studies of the mosquitoes have already been performed, but future research should be done in this direction, to find out if these behaviour differences are due to ecological conditions, including the cultural differences between the villages (ethnic origin, housing, way of living, etc...) and/or if the differences are due to the presence of *An. darlingi *sub-species in the same region.

For the first time in French Guiana, entomological parameters could be related to the malaria transmission patterns. The peak of malaria cases occurring during the dry season is concomitant with the peak in the number of infected mosquitoes surviving enough to transmit (IMT) (Figure 7). This variable is unusual but was chosen to avoid the necessary approximations used in the estimation of the vectorial capacity. The IMT needs four variables and three of them can be extracted from field data, and take into account the mosquito density with the HBR, the survival with the PR, the infection rate and the duration of the extrinsic incubation period (EIP). This last parameter is depending on the temperatures [33,34] and higher temperatures found in French Guiana during the dry season from July to October lead to a shorter EIP duration. Although a reduced mosquito survival has been reported for higher temperatures [51], the mosquito survival estimated from the parity rates is not significantly different along the year. The shorter EIP will cause an increase in the IMT number, and consequently in the transmission risks.

## Conclusion

The entomological investigations carried out in the two villages belongings to two different ethnic groups show clearly that the malaria transmission patterns are strongly different in the year and in the nights according to the location of the village. The numbers of infected mosquitoes surviving enough to transmit peaks in August and is concomitant with the peak of malaria cases and could be thus considered as an indicator of the transmission risk. The results presented herein will help to better understand and estimate the malaria transmission risks along the Maroni River of French Guiana, and must be used to develop better malaria prevention and vector control measures. The actions implemented in the two villages, and more largely in the two ethnic regions must integrate the differences including both cultural habits and mosquito behavior. More precisely, in the village of Loca, malaria transmission peaks from June to August and is much lower from October to May. Consequently, it can be recommended to focus the vector control efforts during the summer months. The transmission risk is also more important during the second part of the night. Because people are living in wooden houses, some practical vector control measures could be house-spraying, fixing of impregnated insect screen to windows and doors, recommendation to use repellents for activities during the second part of the night and, above all, distribution of impregnated bed-nets. In the village of Twenke, the transmission risk remains very high all year long. Consequently, malaria prevention and vector control must be maintained regularly all year round and eventually reinforced during the dry season. The transmission risk is higher during the first part of the night and because the people are living in houses with only one or two walls, the impregnated mosquito bed-nets is the almost exclusive option for the children. For the adults, usually meeting outside during this first part of the night, without protection, the recommendation to use repellents may not be followed (for cultural reasons). Very few options are thus remaining to protect the population, except the use of mass prophylaxis.

Finally, vector studies coupled with epidemiological studies [52,53] are the only approach to propose solutions to prevent, as much as possible, malaria transmission in inland French Guiana. The studies on the behaviour of *An. darlingi *in French Guiana indicate that further investigations on this species are needed, in particular genetic studies, to better understand the differences between the populations at the local and regional level. The use of this knowledge will result in the improvement of malaria prevention in French Guiana and more widely in the Amazons.

## Competing interests

The authors declare that they have no competing interests.

## Authors' contributions

FF have made substantial contributions to the conception and design of the study, participated to the acquisition of data, carried out the analysis and interpretation of the data and was involved in the manuscript redaction. PG have made substantial contribution in the design of the study, the acquisition of the data, carried out the ELISA, and was involved in the verification of the data analysis and the critical revision of the manuscript. RC participated to the design of the study and the acquisition of the data. JI have made substantial contribution in the acquisition of the data and logistics in field. RG was involved in data analysis and interpretation, in drafting the manuscript and revising it critically for important intellectual content. All authors read and approved the final manuscript.

## Supplementary Material

Additional file 1 **Field and transformed data for HBR, PR, Infective Bites, IMT and statistical analysis**. The data provided in a table (.xl format) with several sheets represent all detailed results on the mosquitoes collected during the two-year survey in the two villages of Loca and Twenké, as well as the results of the ELISA on individual mosquitoes and the estimates of Human Biting Rates (HBR), Parity Rates (PR), infective bites and Infected Mosquitoes surviving enough to Transmit (IMT) per month and per hour. The statistical analyses with the Kruskall-Wallis ANOVA are also reported in the Table. Click here for file
